# Menstrual Problems and Lifestyle among Spanish University Women

**DOI:** 10.3390/ijerph17207425

**Published:** 2020-10-12

**Authors:** Elia Fernández-Martínez, Tania Fernández-Villa, Carmen Amezcua-Prieto, María Morales Suárez-Varela, Ramona Mateos-Campos, Carlos Ayán-Pérez, Antonio José Molina de la Torre, Rocío Ortíz-Moncada, Ana Almaraz, Gemma Blázquez Abellán, Miguel Delgado-Rodríguez, Jéssica Alonso-Molero, Virginia Martínez-Ruíz, Agustín Llopis-Morales, Luis Félix Valero Juan, José Mª Cancela Carral, Sandra Martín-Peláez, Juan Alguacil

**Affiliations:** 1 Department of Nursing, University of Huelva, 21071 Huelva, Spain; 2 Research Group on Gene-Environment Interactions and Health (GIIGAS)/Institute of Biomedicine (IBIOMED), University of León, 24071 León, Spain; ajmolt@unileon.es; 3 Department of Preventive Medicine and Public Health, University of Granada, 18016 Granada, Spain; carmezcua@ugr.es (C.A.-P.); virmruiz@ugr.es (V.M.-R.); sandramartin@ugr.es (S.M.-P.); 4 Consortium for Biomedical Research in Epidemiology & Public Health (CIBER Epidemiología y Salud Pública-CIBERESP), 28029 Madrid, Spain; 5 Biosanitary Research Institute ibs., 18014 Granada, Spain; 6 Area of Preventive Medicine and Public Health, Department of Preventive Medicine and Public Health, Food Sciences, Toxicology and Legal Medicine, School of Pharmacy, University of Valencia, 46100 Valencia, Spain; maria.m.morales@uv.es (M.M.S.-V.); agustinllopis@gmail.com (A.L.-M.); 7 Preventive Medicine and Public Health, University of Salamanca, 37007 Salamanca, Spain; rmateos@usal.es (R.M.-C.); luva@usal.es (L.F.V.J.); 8 Well-Move Research Group, University of Vigo, 36310 Vigo, Spain; cayan@uvigo.es; 9 Area of Preventive Medicine and Public Health, Food and Nutrition Research Group, University of Alicante, 03550 Alicante, Spain; rocio.ortiz@ua.es; 10 Preventive Medicine and Public Health, Faculty of Medicine, University of Valladolid, 47005 Valladolid, Spain; aalmaraz@med.uva.es; 11 Departament of Medical Sciences, Faculty of Pharmacy, University of Castilla-La Mancha, 02071 Albacete, Spain; gemma.blazquez@uclm.es; 12 Department of Preventive Medicine and Public Health, University of Jaen, 23071 Jaén, Spain; mdelgado@ujaen.es; 13 CIBERESP, Institute of Health Carlos III, Ministry of Health, 28029 Madrid, Spain; 14 University of Cantabria–IDIVAL, 39011 Santander, Spain; alonsomoleroj@gmail.com; 15 Galicia Sur Health Research Institute (IIS Galicia Sur), Sergas-UVIGO, HealthyFit Research Group, Faculty of Education and Sport Sciences, University of Vigo, 36310 Pontevedra, Spain; chemacc@uvigo.es; 16 Natural Resources, Health and Environment Research Center (RENSMA), University of Huelva, 21071 Huelva, Spain; juan.alguacil@dbasp.uhu.es

**Keywords:** menstrual disorders, diet, lifestyle, university students

## Abstract

Menstrual problems affect many young women worldwide, conditioning both their academic performance and quality of life. This study sought to analyse the prevalence of menstrual problems and their possible relationship with lifestyle among Spanish university women, as part of a research project (UniHcos Project) involving a cohort of 11 Spanish universities with 7208 university students. A descriptive analysis was performed using the bivariate chi-square test and the Student’s *t*-test together with a binary logistic regression, in which the dependent variable was ‘suffering from menstrual problems’. Menstrual problems were identified in 23.8% of the students, representing women who paid more visits to the doctor and to emergency rooms, and who consumed more painkillers and contraceptives. In relation to dietary preferences, menstrual problems were 1.39 (CI 95% 1.22–1.61; *p* = 0.000) times more likely among women classified as high-risk alcohol users according to the AUDIT questionnaire, and 1.187 (CI 95% 1.029–1.370; *p* = 0.019) times greater among those who consumed sweets daily, 1.592 (CI 95% 1.113–2.276; *p* = 0.011) times more frequent among those who eat fish daily, and 1.199 (CI 95% 1.004–1.432; *p* = 0.045) times greater among those who were dieting. Menstrual problems affect many college students and potentially modifiable lifestyle variables exist which may influence their prevalence. It would be interesting to develop programmes to promote women’s health in the university context.

## 1. Introduction

Menstrual problems primarily affect women of childbearing age. Dysmenorrhea or menstrual pain constitutes the main gynaecological complaint for many young women. Previous studies estimate that 60–90% of young university women worldwide suffer from dysmenorrhea, whereas in Spain approximately 75% of women are affected, although exact figures are difficult to calculate as this problem is perceived as being normal [1,2,3]. The literature distinguishes between two types of dysmenorrhea: primary dysmenorrhea, which is not related to any anatomophysiological alteration, and secondary dysmenorrhea, which is associated with a gynaecological problem, notably endometriosis [4]. Endometriosis is defined as the presence of functional endometrial glands and stroma outside the uterine cavity [5], and represents one of the most common benign gynaecological proliferations in premenopausal women, estimated to affect 10–15% of young women [6]. Nonetheless, diagnosis is often delayed and under-diagnosed, thus, endometriosis is frequently confused with primary dysmenorrhea because the main symptom is pain, which often derives in limitations affecting daily life [7]. However, there are other problems related to menstruation, such as menstrual irregularity which is also very prevalent in young women during their first menstrual cycles [8].

Another dysfunction that affects a large number of young women is polycystic ovary syndrome which is defined as an endocrine disorder characterized by hirsutism, anovulation, and polycystic ovaries, which increases the risk of diabetes and metabolic syndrome, among other cardiovascular and metabolic problems [9]. According to various studies, this problem affects between 4–21% of women of childbearing age [10,11,12]. In addition, some gynaecological problems such as endometriosis or polycystic ovary syndrome are also associated with an increase in fertility dysfunctions [13,14].

Menstrual problems affect women’s quality of life, entailing a number of limitations, associated with absenteeism, and a negative impact on academic performance [15,16,17]. Despite this, previous studies have shown that the information that most women receive on this subject stems primarily from family and friends, since the majority of those affected fail to consult professionals because they consider that their symptoms are normal or they feel that professionals may not be able to help. Thus, many women prefer self-care alternatives, even though these are usually not the best choice [18,19]. The main self-care strategy used by these young women is self-medication with non-steroidal anti-inflammatory drugs for the relief of menstrual pain, which has a great economic impact and possible side effects; furthermore, in up to 18% of the cases, their pain fails to respond to this pharmacological approach [19,20].

In addition, diet and lifestyle have been related to different menstrual problems throughout recent years, attributing this relationship mainly to the influence of certain foods and habits on vascular irrigation or the level of oestrogens and prostaglandins [21,22,23,24,25]. Several studies point to the consumption of fruits and vegetables as protective factors for menstrual problems on account of their high vitamin and mineral content [23,26,27,28]. In addition, skipping breakfast and being on a diet for weight loss are considered risk factors [23,29], however, for most habits and foods, such as the consumption of sweets, meat, fish and eggs, the evidence available is limited or contradictory [21,22,23,24,25,26]. Women in different countries have diverse dietary preferences and customs, however in Spain, the scientific evidence on the prevalence of menstrual problems in university girls and lifestyle is limited and published studies include small samples [3]. The present study aimed to analyse the prevalence of menstrual problems among young university women across several Spanish provinces and to examine the association of these with lifestyle factors, such as the consumption of alcohol, tobacco and certain foods.

## 2. Materials and Methods

### 2.1. Design and Participants

A cross-sectional analysis was performed, using a cohort from the UniHcos research project, a multi-centre, multi-purpose prospective cohort study involving 11 Spanish universities (León, Vigo, Jaén, Granada, Salamanca, Huelva, Alicante, Cantabria, Valladolid, Castilla-La Mancha and Valencia) [27]. The main objective of this research project was to learn about the lifestyle habits of students upon entering university and how these are modified during the course of their academic studies.

The main inclusion criteria were students enrolled for the first time as first-year undergraduate students at one of the universities participating in the UniHcos study. Only female students under 35 years of age were included in the present analysis as it was essential for women to be of childbearing age for the purpose of this study. Thus, of 10,167 participants, 7028 (69.13%) were included in the study because they met the inclusion criteria for analysing the proposed objective.

### 2.2. Data Collection Procedure

The study protocol is published elsewhere [27]. Briefly, each student was sent an institutional email as an invitation to voluntarily participate in the study. Informed consent and data were collected through an anonymous self-completed questionnaire via the SphinxOnline^®^ platform (Le Shphinx Developpement, Chavanod, France) during the 2011–2019 study period.

### 2.3. Instrumentation

For the present study, we analysed information on sociodemographic and anthropometric data, variables related to the menstrual problems suffered, diagnoses, drug consumption and activity limitations derived by these problems, as well as information on tobacco consumption, alcohol consumption risk via the Spanish version of the AUDIT questionnaire [28,29], and responses related to food consumption. In relation to alcohol, the cut-off point (AUDIT questionnaire) was considered to be 8 points for considering a high-risk of suffering future related physical and/or psychological damage [28,29]. Food consumption was analysed in relation to women’s daily consumption to facilitate comparison with other studies on gynaecological issues and lifestyle [3,26]. The Body Mass Index (BMI) was calculated from the self-reported weight and height of each participant and interpreted according to the World Health Organization classification: thus a BMI under 18.5 is considered underweight, 18.5–24.99 as normal, 25.0–29.99 being overweight or pre-obese, and 30 or above as obese [30].

### 2.4. Ethical Aspects

The UniHcos project obtained a favourable report from the Ethics Committees (ETHIC-ULE-007-2016) of all the collaborating universities and the database complies with current legislation in Spain on the protection of personal data.

### 2.5. Study Outcome

The main study variable was ‘suffering from menstrual problems’ and was collected in a dichotomous manner by asking: do you suffer from menstrual problems? (yes/no) in the context of a list of health problems on which students were consulted.

### 2.6. Independent Variables

The main independent variables were related to anthropometric, sociodemographic and academic characteristics, limitation of activities of daily living, health consultations, drug consumption and habits regarding lifestyle, daily food and drink consumption.

The following quantitative anthropometric, sociodemographic and academic variables: age, weight, height, BMI and qualitative region, type of access to university, branch of study, limitation of activities of daily living (yes/no) and BMI, categorized according to the WHO.

Regarding the health consultation variables, information was collected on whether a doctor had confirmed a menstrual problem (yes/no), whether women had consulted the doctor in the previous month (yes/no) and whether they had visited an emergency department in the previous year (yes/no).

In relation to the consumption of drugs, information was collected on whether women had taken medication for this problem in the last 12 months (yes/no), the consumption of analgesics in the last two weeks for any ailment (yes/no) and the current consumption of hormonal contraceptives (yes/no). Concerning harmful habits, a variable on alcohol consumption was analysed. Alcohol consumption risk was evaluated using the Spanish version of the AUDIT questionnaire, considering the interpretation described in the literature (consumption risk: yes/no) and another item provided information on whether they were current smokers (yes/no).

For the lifestyle variables, a dichotomous variable gathered whether the respondent exercised 3 h or more per week (yes/no). Likewise, the variables on food consumption were dichotomized for the present study in relation to daily consumption in the line of previous studies [3,31], to enable the comparison of results. Finally, additional diet-related variables were collected, according to the previous studies, such as whether the women ate breakfast every day (yes/no) and whether they were on a diet (yes/no).

### 2.7. Data Analysis

For the analysis of descriptive data, frequencies and percentages were used for the qualitative variables, whereas the mean and standard deviation were used for the quantitative variables. A chi-square test was performed to verify whether there was a relationship between the groups and the Student’s t-test was used to compare mean scores between groups. The logistic regression was used for the dependent variable menstrual problems, including all age-adjusted study variables in the forced model. The level of statistical significance was set at 0.05. All analyses were performed with the IBM-SPSS statistical package, version 20.0 (IBM Corp. Released 2011. IBM SPSS Statistics for Windows, Version 20.0. Armonk, NY, USA: IBM Corp).

## 3. Results

### 3.1. Socio-Demographic Characteristics

The study sample comprised 7208 women from 11 Spanish universities. 38.6% of the participants were from the north of Spain and 61.38% were from Southern Spain. Their mean age was 19.5 ± 12.73 and the majority of women (84%) accessed university from high school. Their socio-demographic characteristics are shown in Table 1 and information regarding their access to university, field of study and lifestyle variables are shown in Table 2.

### 3.2. Menstrual Problems, Socio-Demographic Characteristics and Lifestyle

Of 7208 women consulted, 23.8% (1715) reported suffering from menstrual problems. The mean age of women with menstrual problems was 19.67 ± 2.81 years, compared to 19.46 ± 2.70 years for those without (*p* = 0.007). In relation to the BMI, the mean sample was 21.9 ± 3.5 Kg/m^2^, with no statistical difference between women with and without menstrual problems when comparing mean BMI scores (21.89 ± 3.38 Kg/m^2^ vs 21.92 ± 3.81 Kg/m^2^ respectively, *p* = 0.790). However, when participants were grouped according to WHO categories in relation to BMI, differences between groups were found (*p* = 0.008). Taking into account the WHO BMI categories, a higher prevalence of menstrual problems was identified in obese women with 32.1% (68) of those affected, compared to 23.3% (1213) in underweight, 26% (224) in normal weight, and 22.6% (194) in overweight (Table 3).

Three out of four women who reported having menstrual problems (1284 women, 74.9%) had been diagnosed by a health professional and 62.4% (1070) were currently taking treatment for their menstrual problem.

Of all the students who had a menstrual problem, 34.1% (585) reported having consulted a doctor in the month prior to the interview, compared to 25.5% (1403) of those who did not have this type of problem (*p* = 0.0001). Among patients with menstrual problems, the percentage of those who attended the emergency department in the last 12 months was also higher than that of women without this problem (*p* = 0.0001). A higher proportion of women with menstrual problems reported health-related activity limitations in daily life than women without these problems, even though they had other complaints (*p* = 0.000). Regarding drug use, the proportion of women taking hormonal contraceptives (OCPs) and analgesics was higher among women with menstrual problems (*p* < 0.01, *p* < 0.01).

When comparing the prevalence among women with different BMI (*p* = 0.008), a higher proportion was identified in women with obesity. No differences were found between the proportion of menstrual problems in women from different universities, neither when grouped by communities, nor in two large regions of the country, i.e., north-south (*p* > 0.05). However, differences were found when comparing the prevalence of menstrual problems in women belonging to different branches of study (*p* = 0.000).

### 3.3. Diet and Menstrual Problems

Regarding alcohol consumption, more women at risk were identified among those with menstrual problems (*p* = 0.000). In the group of women with some kind of menstrual problem, more women smoked (33.8%), compared to 28.3% of those who did not have a menstrual problem (*p* = 0.001). Among the women who presented a menstrual problem, 14.1% were dieting versus 11.3% of those without a menstrual problem (*p* = 0.001). Conversely, more women with menstrual problems skipped breakfast (*p* = 0.002), and ate more sweets daily than those without (*p* = 0.016). Also, the proportion of women with menstrual problems was higher among women who ate fish daily compared with those who did not consume fish (*p* = 0.032).

### 3.4. Logistic Regression on Menstrual Problems

The results of the logistic regression are shown in Table 3. Having menstrual problems was 1.40 (CI 95% 1.15–1.71; *p* = 0.001) times more frequent in students of Arts and Humanities and 1.21 (CI 95% 1.04–1.41; *p* = 0.015) times more frequent when compared to students of Health Sciences. Consumption of OCPs was 3.48 (CI 95% 2.05–4.09; *p* = 0.000) times higher among those with menstrual problems than among those without and consumption of analgesics was associated to 1.39 (CI 95% 1,24–1.57; *p* = 0.000) times more cases. Women who had consulted their doctor in the previous month were 1.17 (CI 95% 1.03–1.33; *p* = 0.015) more likely to have menstrual problems, also those who had visited the emergency department in the previous 12 months displayed 1.17 (CI 95% 1.05–1.33; *p* = 0.007) times greater probabilities of having menstrual problems. Those who perceived some limitation of their activity in daily life due to health reasons were also 2.53 (CI 95% 2.24–2.85; *p* = 0.000) times more likely to suffer from menstrual pain.

In relation to diet, menstrual complaints were 1.39 (CI 95% 1.21–1.61; *p* = 0.000) times greater among women classified as high-risk alcohol users according to the AUDIT questionnaire, 1.19 (CI 95% 1.03–1.37; *p* = 0.019) times greater among those who consumed sweets daily, 1.59 (CI 95% 1.11–2.28; *p* = 0.011) times higher among those who ate fish daily, and 1.20 (CI 95% 1.00–1.43; *p* = 0.045) times more likely among those who were dieting.

When classifying all students depending on the area of study, i.e., ‘health sciences’ or ‘other’, students enrolled in studies other than ‘health sciences’ were more likely to report menstrual problems than those studying ‘health sciences’ (OR = 1.23 (1.07–1.42; *p* = 0.003, data not shown)).

## 4. Discussion

Our study reveals a high prevalence of menstrual problems in young university women (23.8%), together with an elevated consumption of analgesics (63.7%) and OCPs (18.5%), and numerous visits to the doctor and emergency services by these women (46%). In addition, approximately half of the women with menstrual problems reported activity limitations in daily life (48.9%).

The prevalence of menstrual problems identified, although high, is lower than that reported in a recent study on dysmenorrhea among Spanish university women [3]. This may be because this study did not specifically inquire about dysmenorrhea, but rather asked participants about menstrual problems, and, as the literature indicates, many women consider dysmenorrhea to be normal and therefore this problem may be underreported if women are not asked directly about dysmenorrhea or menstrual pain [18,32]. Likewise, the present study highlights that approximately 25% of the women had not been diagnosed with a menstrual problem, although they were self-medicating with drugs. As indicated in previous studies, it is common for women not to consult health professionals about their menstrual problems because they consider that they will not be able to help them with their condition, considering that it is a problem affecting other women in their environment, such as family members and friends [1,18,33,34]. The present study identified that more female students enrolled in humanities programmes suffered from menstrual problems compared to women studying science degrees. This may be because women with menstrual problems, and especially those with pain, may tend to opt out of these areas due to the rigors of science training. Furthermore, this could be interpreted as meaning that women who suffer from this problem avoid studying health-related careers. Nonetheless, this is an aspect which should be examined in depth in future studies. The increased use of analgesics and OCPs in the women of our sample who suffered from menstrual problems can be attributed to the fact that the most common problems that affect young women are usually addressed with both these drugs. However, the use of OCPs may indeed be a protective factor for menstrual problems. Nonetheless, these results are difficult to interpret, as this study did not collect any information regarding which OCPs were used, and whether these were taken for contraceptive purposes, to address menstrual problems, or for any other purpose.

In recent years, several studies have analysed the relationship between lifestyle and dietary habits and menstrual disorders, with encouraging results, advising further research on this topic [2,23,35,36,37]. Our results show that the daily consumption of sweets, fish, alcohol consumption and dieting is more frequent among women who have menstrual problems, although our findings do not allow us to establish causal relationships when placed in the context of a cross-sectional study. However, previous studies have pointed out that the consumption of sweets may be a possible risk factor for dysmenorrhea, which may be due to the influence of sugar in the absorption of minerals and vitamins involved in muscle contractility, and therefore, in menstrual pain [38,39]. Sugar consumption also influences insulin resistance and hyperandrogenism in the context of the physiopathology of polycystic ovarian syndrome, increasing the risk of additional metabolic problems [40]. In relation to the risk of alcohol consumption, this was significant, and greater among women with menstrual problems, along the lines of a study by Yesuf et al. in Ethiopian university women and a study by Onieva-Zafra et al. among nursing Spanish students [31,41]. However, other studies have reported inconclusive results relating this type of consumption and menstrual problems [3,41,42]. Although we could consider that menstrual problems themselves could be influencing the consumption of greater amounts of alcohol and this is an issue that should be explored in future studies, some authors, such as Charette et al. have not found any association between menstrual problems, such as menstrual distress, and the consumption of these drinks, attributing consumption primarily to social aspects [43]. Regardless of the reason for consumption, it is worrying that more than 30% of female university students have identified high-risk consumption due to the potential future risks involved, such as cardiovascular and mortality problems [44]. In our study we found a higher proportion of women with menstrual problems among those who did not have normal weight, especially in obese women, along the lines of previous research relating obesity with dysmenorrhea [23,45]. However, this data should be interpreted with caution, as women with polycystic ovary syndrome are often obese, due to the associated endocrine disruption. Being on a diet and low weight were also variables identified as possible risk factors for menstrual problems. Thus, in future studies it would be interesting to specifically examine each type of diet, as the results may be in line with the results reported here, considering that hypocaloric diets tend to lead to vitamin deficiencies. Vitamin deficiencies may be related to menstrual problems according to Bajalan et al. and Jahangir [23,46]. Other types of diet such as vegetarian diets may constitute a protective factor for dysmenorrhea, based on the fact that these diets tend to be lower in fat, with lower oestrogen and high omega-3 [21,47]. The characteristics of the Mediterranean diet, due to its anti-inflammatory potential and contribution to normal weight, has also been identified as being beneficial for some problems such as polycystic ovary syndrome and dysmenorrhea [31,48]. Skipping breakfast was also identified as a lifestyle characteristic that was more common in the women of our study who suffered from menstrual problems. These findings are in agreement with previous research, such as a study by Abu et al. who identified that skipping breakfast increased the risk of dysmenorrhea [24]. Regarding fish consumption, our study showed that daily fish consumption was more frequent in women with menstrual problems, however, previous literature has shown that the consumption of fish oil could be a protective factor for dysmenorrhea and endometriosis due to its potential anti-inflammatory effect attributable to omega-3 [36,49,50,51,52]. Omega-3 supplementation is also being studied in polycystic ovary syndrome, with interesting results [53]. However, to our knowledge, no studies to date have analysed the specific type of fish consumed: e.g., white, blue, fresh, canned...; therefore, it is advisable to continue exploring the type of fish and frequency of consumption in women with different gynaecological problems before issuing more specific recommendations in this regard. Other previous studies, such as those carried out by Jurkiewicz-Przondziono et al. and Bajalan et al., indicated interesting results regarding the consumption of fruits, vegetables and dairy products, as possible protective factors for menstrual problems [23,54]; however, our study found no significant findings in relation to these foods.

### Limitations and Recommendations

This study provides new knowledge about the prevalence of menstrual problems in young Spanish women and their lifestyle characteristics. Moreover, this is the first multicentre study to address this issue among university women in Spain. Although some studies have been conducted in this region, they are scarce and with a limited sample size, compared to the more than 7000 participants in this study [3,31]. However, there are some limitations that should be considered when interpreting the results. First, the cross-sectional design prevents the establishment of causality. In future studies, a longitudinal approach would be interesting to analyse whether some variables that have been associated with menstrual problems exist prior to the problem or are a result of the problem, such as the consumption of analgesics, OCPs, or alcohol. Also, the information was collected in the context of a larger study with various health-related objectives and therefore no specific data were collected on menstrual problems and characteristics, including the intensity of menstrual pain or associated symptoms. We did not gather information on certain variables such as marital status, parity and regularity of sexual relations, which are related to menstrual problems, therefore, it is advisable to take this into consideration for future studies. It would be interesting to follow this line of research by studying the menstrual problems at a multicentre level, specifically, in relation to different types of diets and the consumption of indigenous products from different regions. Furthermore, it is necessary to design and implement educational programs for gynaecological health aimed at the university community and to evaluate their impact on menstrual problems.

## 5. Conclusions

Menstrual problems affect a large number of young university students. These women demand more health and emergency services and consume more painkillers and OCPs to relieve their ailments. In addition, there are some potentially modifiable lifestyle issues that are more frequent in women with these problems, such as hazardous consumption of alcohol, smoking, daily consumption of sweets and fish and being on a diet. It would be interesting to implement health education programs on lifestyle to prevent and improve menstrual problems, given the positive effects that these may have on quality of life.

## Figures and Tables

**Table 1:** Socio-demographic characteristics.

Socio-Demographic Characteristics		*Mean* ± SD	*n* (%)
Age		19.51 ± 2.73	
Region	Granada		2099 (29.1%)
	Valencia		1079 (15%)
	Salamanca		851 (11.8%)
	Vigo		805 (11.2%)
	León		660 (9.2%)
	Alicante		596 (8.3%)
	Valladolid		409 (5.7%)
	Huelva		314 (4.4%)
	Jaén		208 (2.9%)
	Castilla-La Mancha		128 (1.8%)
	Cantabria		59 (0.8%)
BMI (kg/m^2^)	<18.5 kg/m^2^		861 (11.9%)
	18.5–24.99 kg/m^2^		5216 (72.4%)
	25–29.99 kg/m^2^		858 (11.9%)
	≤30 kg/m^2^		212 (2.9%)

(BMI): Body Mass Index.

**Table 2:** Type of access to university, field of study and lifestyle.

Type of Access to University, Field of Study and Lifestyle		*n* (%)
Access to university	Baccalaureate	6056 (84.0%)
	Vocational training	907 (12.6%)
	Other university qualifications	142 (2.0%)
	Entrance test for people > 25 years old	103 (1.4%)
Branch of study	Art and Humanities	951 (13.2%)
	Sciences	1030 (14.3%)
	Health sciences	1733 (24.0%)
	Social sciences	3091 (42.9%)
	Engineering	403 (5.6%)
Did you see a doctor in the last month?	Yes	1988 (27.6%)
	No	5220 (72.4%)
Emergency room consultation in the last year	Yes	2833 (39.3%)
	No	4375 (60.7%)
Hormonal contraceptives (OCPs)	Yes	1051 (14.6%)
	No	6157 (85.4%)
Consumption of Analgesics	Yes	3922 (54.4%)
	No	3286 (45.6%)
Activity limitation in daily life	Yes	2191 (30.4%)
	No	5017 (69.6%)
Exercise 3 h per week or more	Yes	3720 (51.6%)
	No	3488 (48.4%)
Tobacco smoking	Yes	2133 (29.6%)
	No	5075 (70.4%)

**Table 3:** Results of the binary logistic regression analysis for suffering from menstrual problems.

Variables		Menstrual Problem		OR ^a^	CI 95%	*p*-Value
		No *n* (%)	Yes *n* (%)			
Geographical region	North	2097 (75.3%)	687 (24.7%)	1.07	0.95–1.21	0.261
	South	3396 (76.8%)	1028 (23.2%)			
Access to university	Entrance exams	4625 (76.4%)	1431 (23.6%)	Ref. ^b^		0.246
	Vocational training	699 (77.1%)	208 (22.9%)	0.89	0.72–1.10	0.287
	Other university qualifications	101 (71.1%)	41 (28.9%)	1.2	0.78–1.85	0.412
	Entrance test for those over 25 years of age	68 (66%)	35 (34%)	1.33	0.78–2.25	0.295
Branch of study	Health Sciences	1382 (79.7%)	351 (20.3%)	Ref. ^b^		0.019 *
	Sciences	799 (77.6%)	231 (22.4%)	1.15	0.94–1.40	0.174
	Arts and humanities	689 (72.5%)	262 (27.5%)	1.4	1.15–1.71	0.001 *
	Engineering and Architecture	300 (74.4%)	103 (25.6%)	1.18	0.90–1.54	0.241
	Social and political sciences	2323 (75.2%)	768 (24.8%)	1.21	1.04–1.41	0.015 *
BMI	Underweight	4003 (76.7%)	1213 (23.3%)	1.14	0.95–1.36	0.153
	Normal	637 (74%)	224 (26%)	Ref. ^b^		0.096
	Overweight	664 (77.4%)	194 (22.6%)	0.94	0.78–1.13	0.5
	Obesity	144 (67.9%)	68 (32.1%)	1.36	0.99–1.88	0.057
Exercise 3 h per week or more	No	2647 (75.9%)	841 (24.1%)	1	0.89–1.13	0.943
	Yes	2846 (76.5%)	874 (23.5%)			
OCPs	No	5102 (79.2%)	1341 (20.8%)	3.48	2.95–4.09	0.000 *
	Yes	391 (51.1%)	374 (48.9%)			
Consume analgesics	No	2663 (81%)	623 (19%)	1.39	1.24–1.57	0.000 *
	Yes	2830 (72.2%)	1092 (27.8%)			
Consult your doctor	No	4090 (78.4%)	1130 (21.6%)	1.17	1.03–1.33	0.015 *
	Yes	1403 (70.6%)	585 (29.4%)			
Emergency room consultation	No	3449 (78.8%)	926 (21.2%)	1.18	1.05–1.33	0.007 *
	Yes	2044 (72.1%)	789 (27.9%)			
Activity limitation in daily life	No	4141 (82.5%)	876 (17.5%)	2.53	2.24–2.85	0.000 *
	Yes	1352 (61.7%)	839 (38.3%)			
Alcohol:Risk consumption	No	3788 (77.8%)	1082 (22.2%)	1.39	1.21–1.61	0.000 *
	Yes	1705 (72.9%)	633 (27.1%)			
Tobacco	No	3940 (77.6%)	1135 (22.4%)	1.01	0.89–1.16	0.84
	Yes	1553 (72.8%)	580 (27.2%)			
Eats breakfast	No	477 (71.4%)	191 (28.6%)	0.84	0.69–1.02	0.079
	Yes	5016 (76.7%	1524 (23.3%)			
On a diet	No	4875 (76.8%)	1473 (23.2%)	1.2	1.00–1.43	0.045 *
	Yes	618 (71.9%)	242 (28.1%)			
Daily dairy	No	1683 (75%)	561 (25%)	1.19	0.85–1.11	0.676
	Yes	3810 (76.8%)	1154 (23.2%)			
Daily fruit	No	3302 (5.8%)	1057 (24.2%)	1.01	0.89–1.15	0.882
	Yes	2191 (76.9%)	658 (23.1%)			
Daily juices	No	4320 (76.5%)	1330 (23.5%)	1.02	0.88–1.18	0.769
	Yes	1173 (75.3%)	385 (24.7%)			
Daily refreshments	No	5121 (76.4%)	1583 (23.6%)	1.02	0.81–1.29	0.853
	Yes	372 (73.8%)	132 (26.2%)			
Daily sweets	No	4352 (76.8%)	1312 (23.2%)	1.19	1.03–1.37	0.019 *
	Yes	1141 (73.9%)	403 (26.1%)			
Daily vegetables	No	3937 (76.5%)	1210 (23.5%)	1.074	0.93–1.24	0.327
	Yes	1556 (75.5%)	505 (24.5%)			
Daily meat	No	4418 (76.1%)	1389 (23.9%)	0.94	0.81–1.09	0.382
	Yes	1075 (76.7%)	326 (23.3%)			
Daily sausages	No	4756 (75.9%)	1514 (24.1%)	0.85	0.71–1.01	0.066
	Yes	737 (78.6%)	201 (21.4%)			
Daily fish	No	5373 (76.4%)	1662 (23.6%)	1.59	1.11–2.28	0.011*
	Yes	120 (69.4%)	53 (30.6%)			
Daily Legumes	No	5277 (76.2%)	1651 (23.8%)	0.81	0.59–1.11	0.187
	Yes	216 (3.9%)	64 (3.7%)			
Daily eggs	No	5363 (76.2%)	1672 (23.8%)	0.93	0.63–1.37	0.711
	Yes	130 (75.1%)	43 (24.9%)			

OR ^a^ Adjusted Odds ratio: Odds ratio adjusted by age and all variables present in the table; ^b^ Reference category; * < 0.05.
